# Transthoracic two-dimensional xPlane and three-dimensional echocardiographic analysis of the site of mitral valve prolapse

**DOI:** 10.1007/s10554-015-0734-7

**Published:** 2015-08-15

**Authors:** Jackie S. McGhie, Lotte de Groot-de Laat, Ben Ren, Wim Vletter, René Frowijn, Frans Oei, Marcel L. Geleijnse

**Affiliations:** Department of Cardiology, The Thoraxcenter, Erasmus University Medical Center, Room BA 302, ’s-Gravendijkwal 230, 3015 CE Rotterdam, The Netherlands

**Keywords:** Mitral valve, Two-dimensional xPlane echocardiography, Three dimensional echocardiography

## Abstract

This study sought to assess the value of two-dimensional (2D) transthoracic echocardiography (TTE), 2D xPlane imaging and three-dimensional (3D) TTE for the definition of the site and the extent of mitral valve (MV) prolapse. Fifty patients underwent transthoracic 2D, 2D xPlane and 3D echocardiography. With 2D xPlane a segmental analysis of the MV was performed, by making a lateral sweep across the MV coaptation line as seen in the parasternal short-axis view. Inter-observer agreement for specific scallop prolapse was for 2D xPlane excellent (97 %, kappa = 0.94) and for 3D TTE moderate (85 %, kappa = 0.67). The respective sensitivities of standard 2D TTE, 2D xPlane, and 3D TTE for the identification of the precise posterior scallop prolapse were for P1 92, 85, and 92 %, for P2 96, 96, and 82 %, and for P3 86, 81, and 71 %. In total, 5 (8 %) prolapsing MV scallops were missed by 2D TTE, 7 (12 %) by 2D xPlane, and 12 (20 %) by 3D TTE. The sensitivity of 3D TTE was significantly lower than standard 2D imaging (80 % versus 93 %, *P* < 0.05). The extent of P2 prolapse was under or overestimated in 5 patients with 2D xPlane and in 9 patients with 3D TTE. 2D xPlane imaging is an accurate, easy to use (compared to 3D TTE) and easy to interpret (compared to 2D and 3D TTE) imaging modality to study the site and the extent of MV prolapse.

## Introduction

Mitral valve (MV) prolapse (MVP) is one of the most common valvular abnormalities in industrialized countries [1]. The site and extent of the prolapse is essential in defining the suitability for MV repair [2]. Many physicians are of the opinion that two-dimensional (2D) transthoracic echocardiography (TTE) is not reliable enough to provide the surgeon with the essential pre-operative information and consider transesophageal echocardiography (TEE) obligatory. However, it should be recognized that newer technology (beam formers and harmonic imaging) has improved TTE quality and TEE is a semi-invasive imaging technique not totally without procedural risk [3–5]. More recently, three-dimensional (3D) TTE has been developed; a technique that is thought to be able to define more precisely the site and extent of the prolapse in a non-invasive manner [6, 7]. However, 3D imaging requires expertise and suffers from limited temporal and spatial resolution [8]. With the 3D matrix transducer, it is also possible to identify the prolapse site and the extent from multiple 2D xPlane views taken from a standard parasternal short axis view of the MV by simultaneous multiplane imaging (SMPI) [9, 10]. This technique requires less expertise and the spatial resolution is only minimally reduced compared to 3D imaging. Therefore, this study sought to assess the value of 2D TTE, 2D xPlane imaging and 3D TTE for the definition of the site and the extent of MV prolapse in patients that underwent MV surgery.

## Methods

### Study population

Between May 2012 and August 2013, 57 consecutive patients with MVP were referred to our center for surgical MV repair because of isolated severe mitral regurgitation (MR). The institutional review board approved the study and informed consent was obtained from all patients.

Prior to surgery a transthoracic 2D, 2D xPlane and 3D echocardiogram in harmonic imaging was performed using an iE33 ultrasound system (Philips Medical Systems, Best, The Netherlands) equipped with an X5-1 matrix probe composed of 3040 elements, with a 1–5 MHz extended operating frequency range, with the patient in the left lateral decubitus position.

### 2D echocardiography

As recommended, four standard 2D imaging planes were used: the parasternal long-axis and short-axis views and the apical four- and two-chamber views [5].

### 2D xPlane mode

**Fig. 1:**
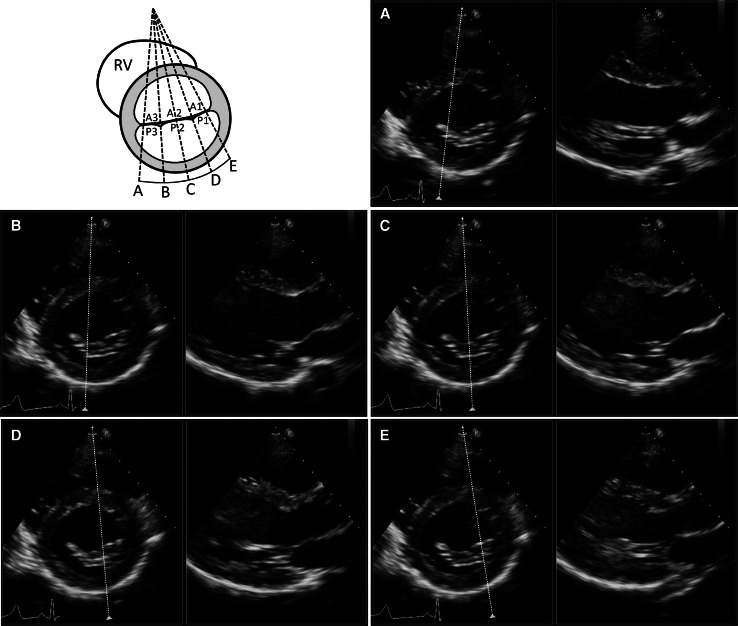
Segmental sweep analysis of the mitral valve scallops with 2D xPlane imaging with lateral tilt. **A**–**E** correspond to the *P*3, *P*2 medial, *P*2 central, *P*2 lateral and *P*1 scallops

**Fig. 2:**
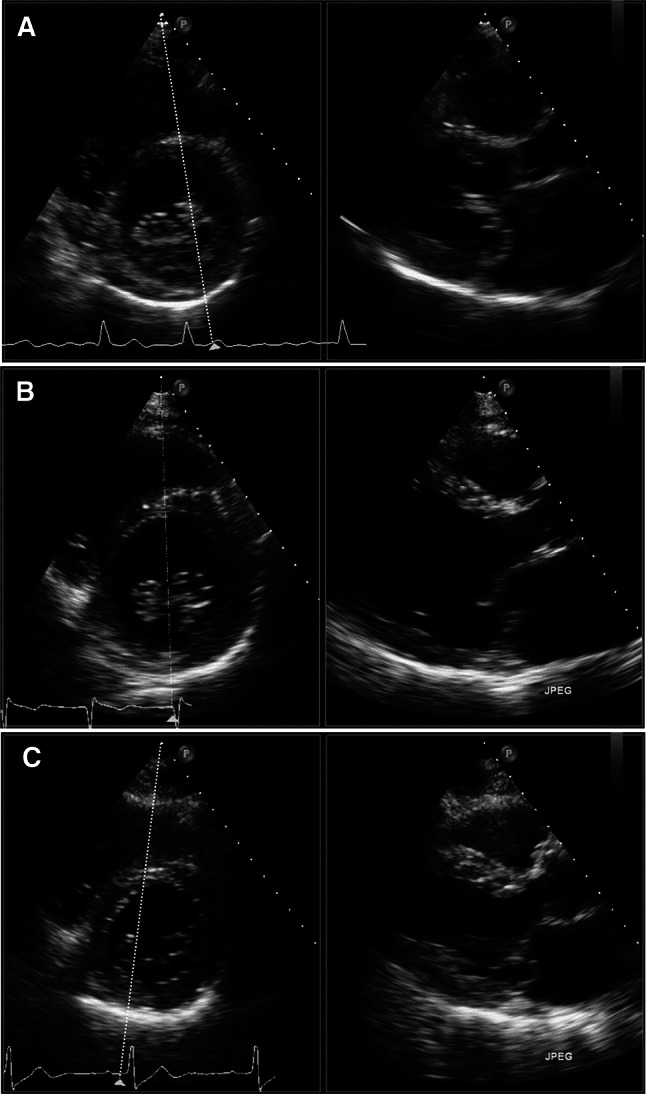
Segmental analysis of the mitral valve scallops with 2D xPlane imaging with lateral tilt. **a**P1 prolapse **b** P2 prolapse **c** P3 prolapse

A segmental analysis of the MV was performed with SMPI in xPlane mode, by making a lateral sweep across the MV coaptation line as seen in the parasternal short-axis view (Fig. 1). In the xPlane mode an orthogonal view can be acquired through the midline of a primary image and displayed as a secondary image. From the midline, additional secondary images can be obtained by a lateral tilt of up to a maximum of +30° to −30° allowing precise visualization of the prolapsing scallop in the secondary image which will resemble a parasternal long axis view. A clear example of a P1, P2 and P3 prolapsing scallop is seen in Fig. 2. The smallest sector able to encompass the mitral valve should be used because in the xPlane mode frame rate will be half of the frame rate of the original image [10]. Mean xPlane frame rate was 37 ± 6 frames per second.

### 3D echocardiography

In patients in sinus rhythm a full-volume data set from four to six R-wave gated sub-volumes during a single end-expiratory breath-hold was acquired and in patients with atrial fibrillation a live 3D data set was acquired to avoid the concerns about stitching artefacts. All full-volume and live 3D data sets were taken from a parasternal or an apical window [11, 12]. The 3D data set was manipulated, off-line, using QLAB version 9 (Philips Medical Systems, Best, The Netherlands) to show an ‘en-face’ or ‘surgical’ view of the MV as seen from the left atrium. The mean 3D volume rate was 36 ± 16 volumes per second.

### Scoring protocol

A senior cardiologist with extensive experience in 2D and 3D echocardiography and MV disease analyzed all echocardiographic data sets blinded to other patient information with at least 10 days between each specific analysis in a random order. MV prolapse and segmental visualization of the affected scallop was classified according to the Carpentier nomenclature [13]. The extent of P2 prolapse was only assessed with 2D xPlane and 3D echocardiography since standard 2D echocardiography is not capable of doing so. The surgical findings served as the gold standard. However, in 6 cases the surgeon only described a P2 prolapse without a clear description on the specific extent of the prolapse. In these 6 cases, intra-operative 3D transesophageal data were used as supplementary gold standard data to describe the extent of the P2 prolapse.

The sensitivity of scoring a P1, P2 or P3 prolapse was calculated as the positive findings of the different modalities divided by the positive surgical findings. The specificity was calculated as the negative findings of the different modalities divided by the negative surgical findings.

The identification of the extent of the P2 prolapse examined with 2D xPlane and 3D TTE was split up into five categories and compared with the surgical finding. The five categories are; Barlow disease (including P1 and P3 prolapse), broad P2 (central, including the centro-medial and centro-lateral edges of the P2 scallop but without P1 or P3 prolapse), small P2 (only central prolapse without incorporation of the centro-medial and centro-lateral edges), asymmetric P2 (central and only one edge) and edge P2 (one centro-medial or centro-lateral part only).

### Statistical analysis

Prolapse site sensitivity and specificity were calculated according to standard formulas.

The degree of inter-observer agreement between the two blinded observers (MLG and JSMcG) for specific scallop prolapse using 2D xPlane and 3D echocardiography was assessed by calculating the Kappa coefficient (a value >0.80 indicating excellent agreement).

## Results

Of the 57 patients referred for surgical repair, 7 patients (12 %) were excluded because a 3D TTE was not possible due to inadequate 2D image quality. In the remaining 50 patients mean age was 61 ± 16 years and 33 (66 %) were men. Forty (80 %) patients were in sinus rhythm and 10 (20 %) in atrial fibrillation. Eleven patients (22 %) had Barlow’s disease involving both the anterior and posterior mitral valve leaflet. In 24 patients (48 %) the prolapse was confined to one or more posterior mitral valve scallops (P1 in 1, P2 in 13, P2 + P3 in 2, P3 in 8 patients). In the remaining 15 patients (30 %) no prolapse was seen and MR was due to mitral annular dilatation with or without retraction in 10 patients (20 %), endocarditis in 3 patients (6 %), and rheumatic disease in 2 patients (4 %).

### Anterior mitral valve scallop

In 16 patients, a prolapsing anterior MV leaflet was seen. In the eleven patients with Barlow disease all prolapsing anterior MV leaflets were recognized with all techniques, apart from one patient in which 3D echocardiography missed the prolapse. In the 5 remaining patients the prolapse was confined to the A2 part in one patient, the A2–A3 part in one patient and the A3 part in three patients. Standard 2D analysis detected anterior MV leaflet prolapse in all patients although distinction between the specific scallops was problematic. 2D xPlane identified the specific prolapse part in all patients where as 3D echocardiography missed the prolapse in two patients with A3 prolapse.

### Localization of posterior mitral valve scallop

**Fig. 3:**
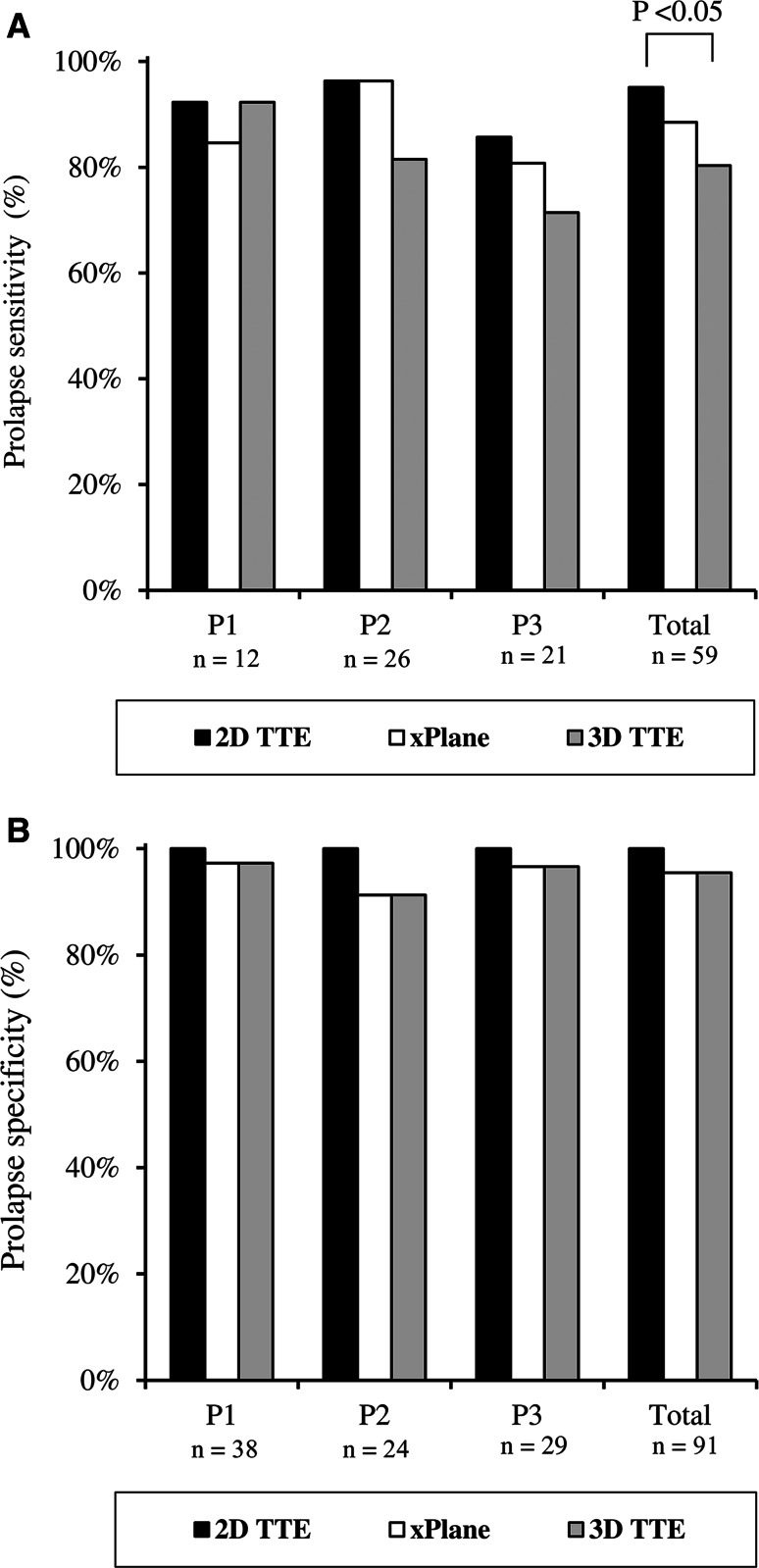
Sensitivity (**a**) and specificity (**b**) for the identification of posterior mitral valve scallop prolapse by the different echo techniques

As seen in Fig. 3a, the respective sensitivities of 2D TTE, 2D xPlane, and 3D TTE for the identification of the precise posterior scallop prolapse were for P1 92, 85, and 92 %, for P2 96, 96, and 82 %, and for P3 86, 81, and 71 %. In total, 5 (8 %) prolapsing MV scallops were missed by 2D TTE, 7 (12 %) by 2D xPlane, and 12 (20 %) by 3D TTE. The sensitivity of 3D TTE was significantly lower than standard 2D imaging (80 vs. 93 %, *P* < 0.05). As seen in Fig. 3b, the respective specificities of 2D TTE, 2D xPlane, and 3D TTE for the identification of the precise posterior scallop prolapse were for P1 100, 97, and 97 %, for P2 100, 91, and 91 %, and for P3 100, 97, and 97 %.

### Identification of the extent of the P2 prolapse

The results of 2D xPlane and 3D TTE for accurately diagnosing the extent of the P2 prolapse are shown in Table 1. All 11 patients with a Barlow MV (that is involvement of the complete anterior and posterior MV leaflet) were correctly diagnosed by both modalities. Seven patients had a broad P2 prolapse. With 2D xPlane 4 were identified correctly and in 3 patients, a prolapsing P2 was seen, but the extent of prolapse was to some extent underestimated (one edge was missed). Whereas with 3D TTE, one was missed completely and 3 were underestimated (in two patient’s one edge was missed and in one patient only a prolapsing edge was identified). Five patients had a small central P2 prolapse, 2D xPlane identified three correctly, overestimated one (that is one edge was also scored as prolapsing) and missed one. 3D TTE identified two correctly and missed three. In the three patients with asymmetric P2 prolapse (center and one edge), all three were correctly diagnosed with 2D xPlane, but two were underestimated (only a prolapsing edge was identified in one patient and only a prolapsing central part in one other patient) with 3D TTE. One patient had a P2 one edge only prolapse that was diagnosed correctly with 2D xPlane and missed by 3D TTE. So, in total 4 and 9 scallop parts were missed or underestimated with 2D xPlane and 3D, respectively.

**Table 1:**
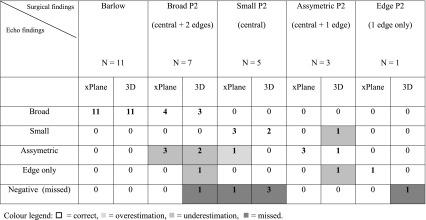
Identification of the extent of P2 prolapse with transthoracic xPlane and 3D echocardiography

### Inter-observer agreement

**Fig. 4:**
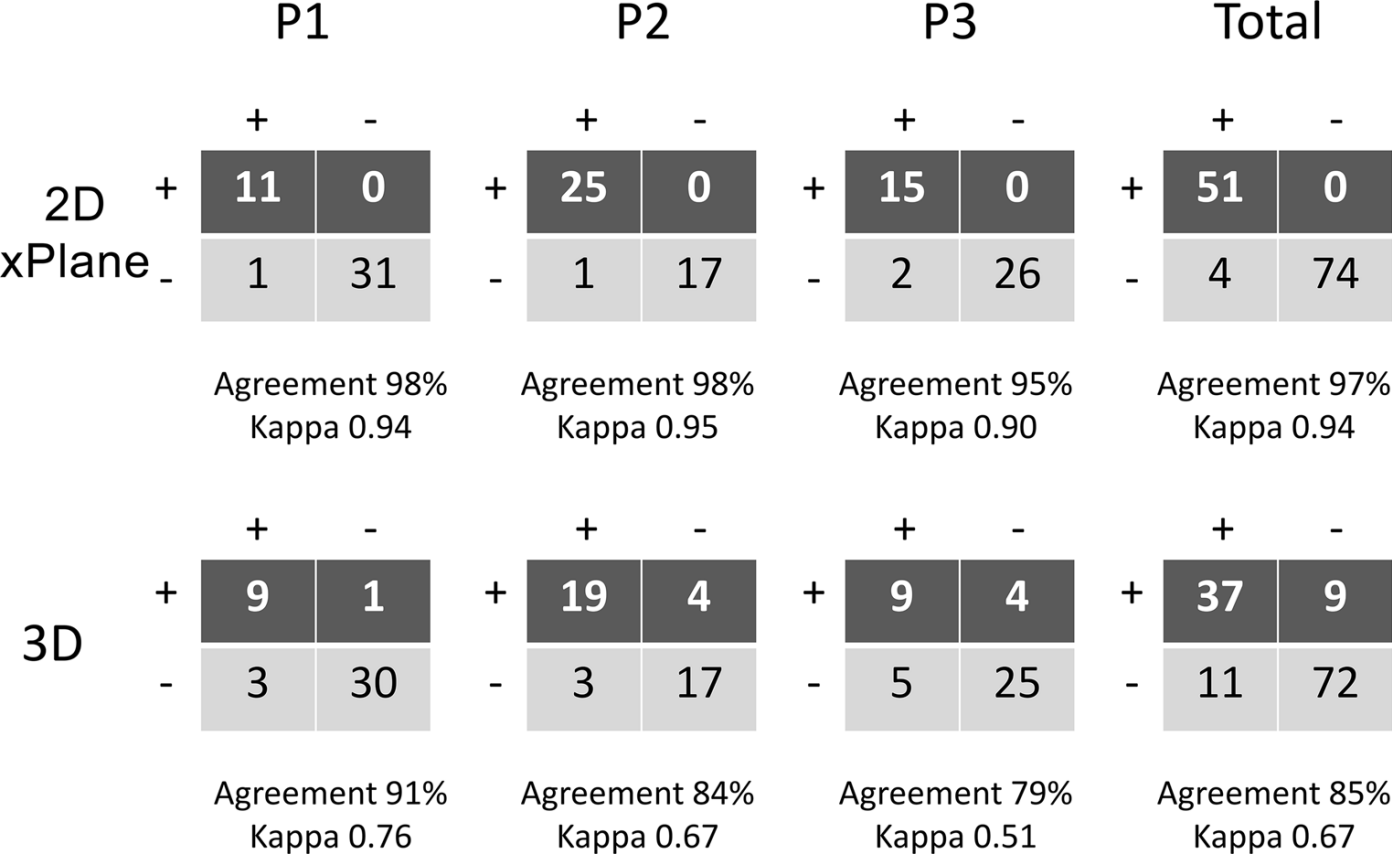
Interobserver variability in assessment of posterior mitral valve scallop prolapse by 2D xPlane and 3D echocardiography

Seven additional 3D TTE were excluded because the second observer determined the 3D quality too poor to reliably assess the site of MV prolapse. Inter-observer agreement for specific scallop prolapse is shown in Fig. 4. For 2D xPlane the agreement was excellent (97 %, kappa = 0.94). For 3D TTE the agreement was moderate (85 %, kappa = 0.67).

## Discussion

In this study we sought to assess the relative value of transthoracic standard 2D imaging, 2D xPlane imaging, and 3D imaging for the definition of the site and extent of MV prolapse. The main results of the study are (1) transthoracic 2D imaging has excellent diagnostic value in detection of the prolapsing MV scallop, (2) 2D xPlane and 3D imaging do not improve detection of the prolapsing MV scallop, (3) the extent and asymmetry of P2 prolapse can, however, only be assessed by xPlane and 3D imaging, and (4) 2D xPlane imaging may be superior to en-face 3D imaging in this latter aspect because it is (a) easier to implement as it is a 2D technique, (b) has a better interobserver agreement, and (c) misses less prolapsing MV scallop parts because of less artifacts (dropouts and side-lobe artifacts) and better spatial resolution.

The definition of the site and extent of MV prolapse plays a crucial role not only in surgical referral but also for the operative plan since different pathology require different levels of surgical expertise based on the complexity of lesions seen with echocardiography [14, 15]. Some authors have reported poor sensitivities of 2D TTE for the identification of prolapse [16], and in particular of the not centrally-located P1 and P3 scallops [4, 17, 18]. Minardi et al. reported sensitivities of 64, 99 and 50 % for respectively P1, P2, and P3 scallop prolapse (although they claimed overall sensitivity was excellent since the middle scallop P2 “represents almost the totality of prolapses”). Also, Beraud et al. reported a correct description in only 22 % in patients with a prolapse other than isolated P2 prolapse. Pepi et al. reported a sensitivity of 40 % for the antero-lateral commissure and 54 % for the postero-medial commissure.

In contrast, Monin et al. reported sensitivities of 95 and 93 % for respectively the central P2 prolapse and the not centrally-located P1 and P3 scallops based on a similar 2D analysis. Our results of sensitivities of 92, 96, and 86 % for respectively P1, P2, and P3 prolapse are in line with these results of Monin et al. Of note, like in our study the echo studies were performed by dedicated “senior” sonographers and analyzed by a cardiologist with extensive experience in MV assessment. As a result of the excellent transthoracic 2D diagnostic results 2D xPlane and 3D imaging was not able to add diagnostic value. On the contrary, 2D-xPlane imaging and in particular 3D imaging resulted in more false negative results.

In the literature there is some controversy over the accuracy of 3D TTE for the evaluation of the site and extent of the MV prolapse. All investigators stated that 3D TTE is a feasible technique in the majority of patients. This was confirmed in our study as in only 7 patients (12 %) the 3D images were deemed not possible because of image quality by the sonographer and in another 7 patients the 3D image quality was found to be inadequate for analysis by one of the two observers. Several investigators stated that the accuracy of 3D TTE for the identification of scallop prolapse is high [7, 17–20] and may even be superior to 2D TTE [17–19] or even 2D TEE [7]. Although Gutiérrez-Chico already noted imperfect results for the not centrally-located (lateral and medial) scallops [20], Zekry et al. pointed out clearly the difficulty in using 3D TTE to localize mitral valve segmental disease especially for the not centrally-located scallops: sensitivities were 7, 93, and 29 % for respectively P1, P2, and P3 scallop prolapse [21]. In our 3D study the sensitivity for the detection of P3 prolapse was also somewhat lower. Of note, Agricola et al. and Beraud et al. used a combination of en-face “surgical” views and 3 to 5 reconstructed longitudinal views (“representing the A1–P1, A2–P2, A3–P3 scallops and the two commissures”) and it was claimed to be, in particular, helpful in patients with commissural prolapse, although results were still sub-optimal.

The identification of 3D volume-rendered images may be difficult even for the experienced observer since a prolapsing scallop should be identified as a convexity or bulge, and often as a bright area when compared with the rest of the mitral valve. Despite exclusion of patients with poor echocardiographic images, the current spatial and temporal resolution of 3D transthoracic transducers in our opinion still limits the interpretation of images. This was evidenced not only by the 12 missed prolapsing scallops (compared to 5 and 7 with respectively 2D TTE and 2D xPlane), but also by the underestimation of P2 scallop extent by 3D.

### 2D xPlane imaging

With the introduction of 2D xPlane imaging it is possible to identify not only a prolapsing MV leaflet but also to assess, like 3D imaging, in a systematic manner the extent of MV prolapse. It is important to realize that the xPlane technique in fact mimics the 3D multiplane reconstruction with as opposite to 3D imaging only a minimal impact on spatial resolution compared to standard 2D imaging with a 2D transducer. Compared to the surgical findings 2D xPlane was a sensitive technique (overall 2D xPlane sensitivity 88 vs. 80 % for 3D) to identify MV prolapse and the inter-observer agreement for identification of the prolapsing MV scallop was excellent. Also, the sensitivity of 2D xPlane imaging for the identification of MV prolapse was not lower than standard 2D imaging, whereas 3D TTE was significantly lower compared to standard 2D imaging. In addition, good results were seen in the identification of the extent of P2 prolapse.

Any sonographer will be able to perform an accurate, rapid, online segmental analysis of the entire coaptation line of the MV with xPlane imaging. Virtually the images do not suffer from a loss in spatial resolution and rhythm irregularities will not affect the data. Although in the present study the identification of MV prolapse presence was not superior to standard 2D imaging it should be realized that the 2D images were interpreted by a senior cardiologist highly experienced in MV evaluation.

### Clinical implications

Although not discussed in this article, 2D and 3D TEE imaging are excellent imaging tools to describe the MV geometry and mechanism of regurgitation and to guide the surgical approach. However, TEE is a semi-invasive imaging technique not totally without procedural risk. [3, 22] Because 2D xPlane is an easy, accurate and noninvasive imaging modality we suggest standard 2D supplemented with 2D xPlane to be used in the outpatient clinic for optimal assessment of MV geometry and mechanism of regurgitation. Only in the few patients in whom doubt persists (in particular the involvement of the para-commissural scallops) 2D and/or 3D TEE imaging should be performed in the outpatient’s clinic. Finally, pre-operative TEE in the operating room (the ideal circumstance for studying the geometry and mechanism of the MV) may further refine the diagnosis and guide the surgical approach.

## Limitations

The spatial resolution of the X5-1 matrix transthoracic probe remains somewhat inferior to the stand-alone 2D transducer and in addition the frame rate (temporal resolution) drops by half when entering the xPlane mode. This drop in temporal resolution however, does not seem very important in the assessment of the site and extent of MV prolapse and can be brought to a minimum by ensuring that the smallest sector able to encompass the MV is used.

Care must be taken with the interpretation of the extent of prolapse from the standard parasternal short axis view analysis since the motion of the heart throughout the cardiac cycle may result in the reference line not transecting the same region of interest at any time point in the heart cycle.

The echo studies were performed by dedicated “senior” sonographers and analyzed by a cardiologist with extensive experience in MV assessment. Therefore, our results may not be generalized to less experienced centers. In addition, TEE imaging was not considered in the design of the study because the aim of the study was to assess the relative value of transthoracic standard 2D imaging, transthoracic 2D xPlane imaging, and transthoracic 3D imaging for the definition of the site and extent of MV prolapse defined by the surgical standard.

Finally, the anatomical findings at surgery served as the gold standard. It should be recognized that surgeons assess an immobile valve in a flaccid heart whereas echocardiography assesses a dynamic valve. Unfortunately, there is no practical alternative to this approach.

## Conclusion

2D xPlane imaging is an accurate, easy to use (compared to 3D TTE) and easy to interpret (compared to 2D and 3D TTE) imaging modality to study the site and the extent of MV prolapse.
